# Identification of a spontaneously arising variant affecting thermotaxis behavior in a recombinant inbred *Caenorhabditis elegans* line

**DOI:** 10.1093/g3journal/jkad186

**Published:** 2023-08-12

**Authors:** Jihye Yeon, Charmi Porwal, Patrick T McGrath, Piali Sengupta

**Affiliations:** Department of Biology, Brandeis University, Waltham, MA 02454, USA; Department of Biology, Brandeis University, Waltham, MA 02454, USA; School of Biological Sciences, Georgia Institute of Technology, Atlanta, GA 30332, USA; Department of Biology, Brandeis University, Waltham, MA 02454, USA

**Keywords:** *Caenorhabditis elegans*, recombinant inbred line, thermotaxis, *ttx-1*

## Abstract

Analyses of the contributions of genetic variants in wild strains to phenotypic differences have led to a more complete description of the pathways underlying cellular functions. Causal loci are typically identified via interbreeding of strains with distinct phenotypes in order to establish recombinant inbred lines (RILs). Since the generation of RILs requires growth for multiple generations, their genomes may contain not only different combinations of parental alleles but also genetic changes that arose *de novo* during the establishment of these lines. Here, we report that in the course of generating RILs between *Caenorhabditis elegans* strains that exhibit distinct thermotaxis behavioral phenotypes, we identified spontaneously arising variants in the *ttx-1* locus. *ttx-1* encodes the terminal selector factor for the AFD thermosensory neurons, and loss-of-function mutations in *ttx-1* abolish thermotaxis behaviors. The identified genetic changes in *ttx-1* in the RIL are predicted to decrease *ttx-1* function in part via specifically affecting a subset of AFD-expressed *ttx-1* isoforms. Introduction of the relevant missense mutation in the laboratory *C. elegans* strain via gene editing recapitulates the thermotaxis behavioral defects of the RIL. Our results suggest that spontaneously occurring genomic changes in RILs may complicate identification of loci contributing to phenotypic variation, but that these mutations may nevertheless lead to the identification of important causal molecules and mechanisms.

## Introduction

Animals must adapt their behaviors in response to environmental changes for optimal survival. Forward and reverse genetic approaches in lab-derived or domesticated strains have been extremely powerful in identifying genetic loci associated with behaviors, but the analysis of only laboratory reference strains is insufficient to identify genetic variants driving adaptive evolution in diverse natural environments (Sterken et al. 2015; Niepoth and Bendesky 2020; Jourjine and Hoekstra 2021). Characterization of phenotypic traits in wild strains and causal association of these traits with underlying genetic variation have begun to provide a deeper and richer understanding of the genetic architecture underlying nervous system function and behavioral adaptation (de Bono and Bargmann 1998; Félix and Duveau 2012; Baldwin et al. 2014; Bendesky et al. 2017; Seeholzer et al. 2018; Andersen and Rockman 2022). This approach has been widely used in multiple experimental systems to identify a range of molecules and mechanisms that represent substrates for evolutionary change to modulate neuron and circuit properties.

A simple analysis to identify loci that quantitatively contribute to trait variation involves the interbreeding of 2 strains exhibiting distinct phenotypes to generate a panel of recombinant inbred lines (RILs) (Rockman and Kruglyak 2008; Pollard 2012). The generation of RILs homozygous for different combinations of parental alleles requires growth for multiple generations. Even under controlled laboratory conditions, prolonged cultivation increases the probability of spontaneously arising genetic changes in these RILs (Cook et al. 2006; Zhao et al. 2020). Consequently, the genomic architecture of RILs may not only represent a mosaic of parental alleles but may also include new variants that arose during the process of RIL generation. Although these novel variants complicate analyses of the contributions of parental genotypes to the trait under study, these changes have nevertheless allowed for the identification of relevant genetic changes that also influence phenotypic differences (Cook et al. 2006; Zhao et al. 2020).

*Caenorhabditis elegans* provides a particularly robust experimental system in which to study how genetic changes contribute to natural phenotypic variation (Félix and Braendle 2010; Frezal and Felix 2015). Wild inbred *C. elegans* strains have been isolated from different regions around the world, and their genomes have been sequenced (Andersen et al. 2012; Cook et al. 2016; Cook et al. 2017). Analyses of quantitative trait loci (QTLs) contributing to behavioral variation between the N2 laboratory reference and wild strains have led to a detailed understanding of the genetic basis of natural variation and have allowed for the identification and characterization of new pathways underlying phenotypic and developmental adaptations (Evans et al. 2021).

*Caenorhabditis elegans* is highly sensitive to temperature changes in its environment and exhibits complex and experience-dependent navigation behaviors on spatial thermal gradients (Hedgecock and Russell 1975; Ryu and Samuel 2002). Thermotaxis behavior is driven primarily, although not exclusively, by the single AFD thermosensory neuron pair which responds with high sensitivity to temperature changes in an experience-dependent manner (Mori and Ohshima 1995; Kimura et al. 2004; Clark et al. 2006). A subset of wild *C. elegans* strains has been reported to exhibit differences in their temperature preferences on thermal gradients, but these preferences remain experience-dependent (Anderson et al. 2007; Jurado et al. 2010; Anderson et al. 2011; Gaertner et al. 2012). The analysis of the genetic variants contributing to behavioral differences in thermal preference between 2 *C. elegans* strains suggested complex epistatic interactions among multiple loci whose molecular identities have not been identified (Gaertner et al. 2012).

Here, we examine thermotaxis in a panel of *C. elegans* strains and identify a strain that shows enhanced thermotaxis behavior. In the course of generating RILs to identify the genetic changes leading to this behavioral difference, we identified an RIL that is atactic and also exhibits defects in locomotory behavior. The thermotaxis behavioral defect in this RIL arises due to variants in at least 2 independently segregating loci. We identify 1 of these loci as the *ttx-1* Otx homeodomain gene that acts as a terminal selector factor for the AFD thermosensory neurons (Satterlee et al. 2001). We show that these variants reduce *ttx-1* function and result in both locomotory and thermotaxis defects and that these changes are not present in either parental strain. Our results suggest that spontaneously arising genetic changes in the course of RIL generation can identify loci critical for driving variation in complex phenotypic traits.

## Materials and methods

### Strains

All nematode strains were grown on *E. coli*OP50 and cultivated at 20°C. Complete lists of all analyzed strains and strains generated in this work are provided in Supplementary Tables 1 and 3, respectively. Transgenic strains containing *gcy-8*p::*gfp* in extrachromosomal arrays were generated using PSAA685 (*gcy-8*p::*gfp*) at 5 ng/μl with the *unc-122*p*::dsRed* coinjection marker at 50 ng/μl. All behavioral assays were performed using adult animals that were grown with ample bacterial food for at least 3 generations.

### Generation of RILs

RILs were generated from a cross between CC1 and N2 males carrying the *kyIs37*(*str-2*p*::gfp*) marker. Thirty F2 hermaphrodites from a single heterozygous F1 from this cross were individually placed on plates and allowed to self-fertilize for 8 generations (see Supplementary Fig. 1a). A single hermaphrodite was transferred in each generation to obtain 30 RILs. A similar approach was used to generate RILs from PY12237. Ten F2 progeny from a cross between PY12237 and N2 were placed on individual plates and allowed to self-fertilize for 8 generations.

### Whole-genome sequencing and analysis

Animals were grown on 10-cm High Growth Medium plate (HGMA) until the bacterial food was nearly depleted. Genomic DNA was isolated using the Qiagen DNeasy Blood & Tissue kit (catalog number: 69504). Preparation of DNA libraries and whole-genome sequencing was performed by BGI (www.bgi.com). Each genome was sequenced at ∼30× coverage.

### Sequence analysis

The reads were aligned to the WS260 reference genome using Burrows–Wheeler Aligner (BWA v0.7.17 (Li and Durbin 2009). BAM files were deduplicated and processed using SAMtools v1.9 (Li et al. 2009) and Picard (http://broadinstitute.github.io/picard/). Single-nucleotide variations (SNVs were called by Freebayes and annotated by SnpEff (Cingolani et al. 2012; Garrison and Marth 2012). Variant analyses sequence files can be found at Figshare (https://doi.org/10.6084/m9.figshare.23565756). Custom Python scripts (https://github.com/ptmcgrat/Sequencer/blob/master/NGS_Nematode.py) using the pysam library (https://github.com/pysam-developers/pysam) were used to identify regions of the genome with a large number of clipped and chimeric reads. Read depths were visualized using Integrative Genomics Viewer (IGV (Robinson et al. 2011).

### CRISPR/Cas9-mediated genome editing

To generate *ttx-1(oy184)*, a donor oligonucleotide (IDT: Integrated DNA Technologies) containing the ATG to ACG change along with 25 bp homology arms and a mutated PAM site (TCC to TGC) was injected along with sgRNA (100 ng/μl; IDT) and Cas9 (25 ng/μl; IDT) into N2. *dpy-10* crRNA (20 ng/μl; IDT) was used as the co-CRISPR injection marker (Arribere et al. 2014). F1 animals with Rol and Dpy phenotypes were isolated, and genome editing was confirmed via sequencing. F2 progeny were further screened for homozygosity.

To generate *Y102A11A.1(oy185)*, a donor oligonucleotide (IDT) containing the CAA (Gln) to TAA (STOP) change along with 35 bp homology arms was injected along with crRNA (100 ng/μl; IDT), tracrRNA (100 ng/μl; IDT) and Cas9 (25 ng/μl; IDT), and *unc-122*p::*dsRed* (50 ng/μl) as the coinjection markers into N2. F1 animals with *unc-122*p::*dsRed* fluorescence were isolated and confirmed via sequencing. F2 progeny were further screened for homozygosity.

Sequences of sgRNA and donor oligonucleotides:

sgRNA [*ttx-1(oy184)*]: 5′- CCT//TGACGTCTTCCTCAGCTCCA -3′

Donor oligonucleotide [*ttx-1(oy184*)]: 5′- TCATTTGTCATATATTCGTGCTTCACGTCCTTGACGTCTTCCTCAGCTCCAT -3′

crRNA [*Y102A11A.1(oy185*)]: 5′- CCT//CCTCAGTTGTTCAATCAACA -3′

Donor oligonucleotide: [*Y102A11A.1(oy185*)]: 5′- CCCTCCGGCTGCTCAGCCAGCTCCACAATAAATTATGATACCTCCTCAGTTGTTCAATCAACAAATGCCTCAATTACAAAACCCACAGCAACACAATC -3′.

### Thermotaxis behavior

Thermotaxis behavioral assays were performed as described previously (Takeishi et al. 2020). In brief, a thermal gradient (ranging from 23 to 28°C at 0.5°C/cm) was established on 10-cm NGM agar plates using Peltier thermoelectric temperature controllers (Oven Industries). Fifteen animals grown at 20°C were placed at the center of the assay plate at the start of the assay. The temperature of the edges of NGM agar was measured at the beginning of the assay with a 2-probe digital thermometer (Fluke Electronics). Animal behavior was recorded at a rate of 1 Hz for 35 min using PixeLink CCD cameras controlled by custom written scripts in MATLAB. Videos were analyzed using WormLab (MBF Bioscience) and custom written scripts in MATLAB as described previously (Beverly et al. 2011; Yu et al. 2014).

### Exploration assay

The grid exploration assay was performed essentially as described previously (Flavell et al. 2013). The day prior to the assay, a well-fed young adult animal was picked onto a 35-mm agar plate uniformly seeded with 100 μl of *Escherichia coli*OP50. After 16 h, the animal was removed, and the plate was superimposed on a grid containing 3.5-mm squares. The number of squares entered by the worm as assessed by the presence of tracks in the bacterial food was manually counted. Twenty animals were tested per data point for each genotype.

### Chemotaxis behavior

Chemotaxis behavioral assays were performed as described previously (Bargmann et al. 1993). Well-fed young adult animals were washed twice with S-Basal twice and once with water and placed at the center of a 10-cm NGM agar plate. One μl of the diluted odorant in ethanol or ethanol alone were placed on either end of the plate along with 1 μl of 1 M sodium azide. Chemicals used were isoamyl alcohol (Fisher A393-500) and diacetyl (Sigma B85307). Animals at the odorant, diluent, and throughout the plate were counted after 1 h.

### Fluorescence reporter quantification

Well-fed young adults grown at 20°C were paralyzed with 10 mM levamisole and mounted on 3% agarose pads on a glass coverslip. Animals were examined using a ×63 oil objective (NA 1.4) on a Zeiss Axio Imager M2 epifluorescence microscope. *z*-stack images of individual animals were acquired using Zeiss Pro software (resolution, 1280 × 1024 pixels; exposure time, 120 ms; step size, 0.25 μm). Images were processed in ImageJ (NIH). An ROI was drawn around a single AFD soma per animal, and the fluorescence intensities in 5 optical slices per animal were quantified and averaged. To account for expression variation from extrachromosomal arrays carrying *gcy-8*p*::gfp*, 2 independent transgenic lines were examined for each strain; expression levels were similar between lines.

### Statistical analyses

Statistical analyses were performed using Prism v9.5.1. Comparisons to N2 were performed with *t*-test or 1-way ANOVA. Corrections for multiple comparisons were performed using Tukey's or Bonferroni tests. The statistical test used is indicated in the legend to each figure.

## Results

### The CC1 *C. elegans* strain exhibits enhanced negative thermotaxis behavior

**Fig. 1.**
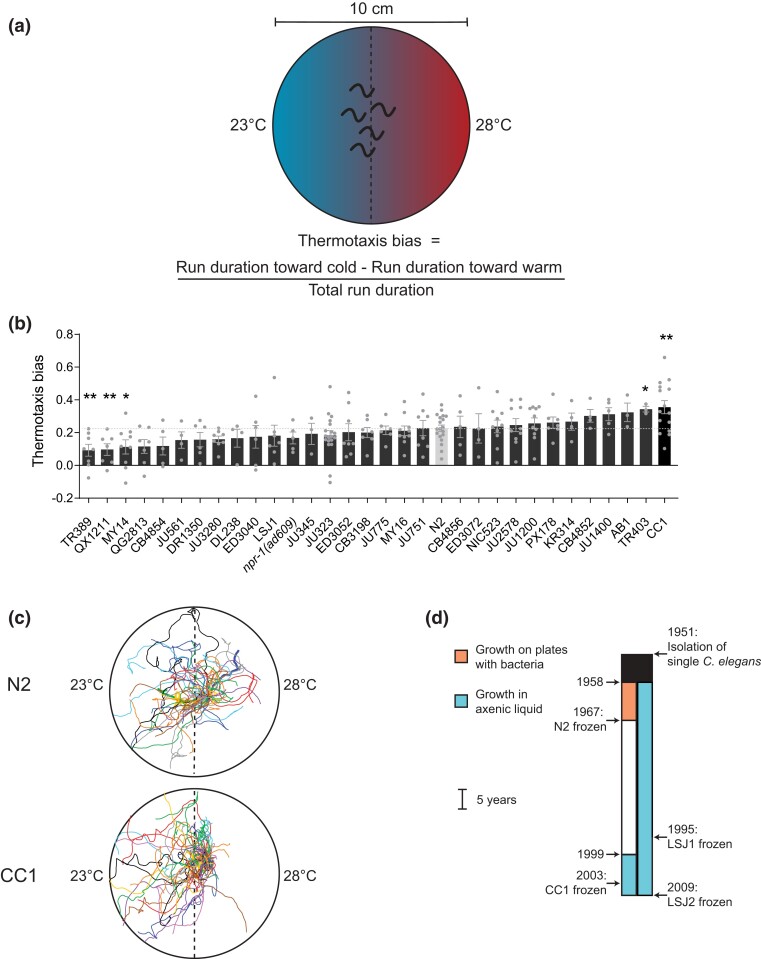
CC1 exhibits improved negative thermotaxis behavior. a) Cartoon of the thermotaxis behavioral assay. Animals grown at 20°C are placed at the center (dotted line) at the start of the assay (see Materials and Methods for additional details). b) Mean thermotaxis bias of shown strains. Each dot is the thermotaxis bias of a single assay with 15 animals. Data shown are from at least 3 independent experiments. Animals were grown at 20°C and assayed on a 0.05°C/cm gradient from 23 to 28°C (a). Errors are SEM. * and ** indicate different from N2 at *P* < 0.05 and 0.001, respectively (*t*-test). c) Representative trajectories of N2 and CC1 animals in a single thermotaxis assay. Trajectories of individual animals are color coded. d) Isolation and growth history of N2, CC1, LSJ1, and LSJ2. Modified from McGrath et al. (2011).

To assess whether wild *C. elegans* strains collected from multiple locations across the world or different laboratory N2-derived strains exhibit variations in thermotaxis behavior, we grew a panel of 30 strains (Supplementary Table 1) (Cook et al. 2017; Lee et al. 2021) at 20°C and examined their behavior on a spatial thermal gradient ranging from 23 to 28°C (Fig. 1a. Since the laboratory N2 strain contains a gain-of-function allele of the *npr-1* neuropeptide receptor as compared with the allele present in wild *C. elegans* strains (de Bono and Bargmann 1998), we also examined the behavior of *npr-1(ad609)* loss-of-function mutants. Under these conditions, as expected, the N2 laboratory reference strain moved toward the colder side of the gradient (negative thermotaxis; Fig. 1b). The negative thermotaxis behavior of the majority of examined strains was not significantly different from that of N2, with the exception of a subset of strains that exhibited decreased negative thermotaxis (Fig. 1b). In contrast, the CC1 strain exhibited significantly enhanced negative thermotaxis (Fig. 1b–c). The velocity of CC1 animals was similar to that of N2 on an isothermal plate (N2, 136.9 ± 3.2 μm/s; CC1, 129.5 ± 7.2 μm/s; 15 animals each at 20°C). CC1 is an N2-derived strain that was frozen following 4 years of growth in liquid axenic media (Fig. 1d) (Szewczyk et al. 2003; McGrath et al. 2011). An additional strain (LSJ1) that was grown in axenic media for >30 years before being frozen down (Fig. 1d) (McGrath et al. 2009; McGrath et al. 2011; Sterken et al. 2015) did not exhibit a similar behavioral phenotype (Fig. 1b), suggesting that the genetic changes in CC1 leading to enhanced negative thermotaxis are unlikely to have arisen due to selective pressure upon growth in liquid acting directly on this trait.

### Enhanced negative thermotaxis behavior in CC1 is unlikely to have arisen due to a loss-of-function mutation at a single locus

**Fig. 2.**
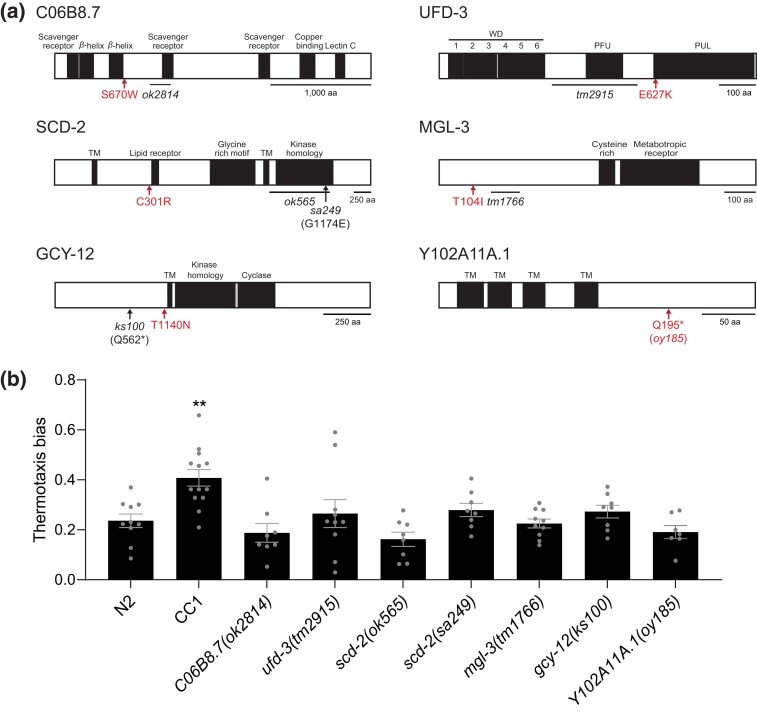
Enhanced negative thermotaxis in CC1 may not be due to loss of function of a single gene. a) Molecular identities and locations of missense variants (red in protein-coding genes present in CC1 but not in N2. Predicted structural domains in each of these proteins are indicated. The extent of the deletions and the location of nonsense mutations in putative null alleles examined for effects on thermotaxis behavior in (b) are shown. The Q195* change in *Y102A11.1* present in CC1 was engineered in the N2 background to generate *oy185*. TM, transmembrane domain. b) Mean thermotaxis bias of N2 animals carrying the indicated mutations. Each dot is the thermotaxis bias of a single assay with 15 animals. Data shown are from at least 3 independent experiments. Animals were grown at 20°C and assayed on a 0.05°C/cm gradient from 23 to 28°C. Errors are SEM. ** indicates different at *P* < 0.01 compared with N2 (*t*-test).

To identify causal changes underlying the improved negative thermotaxis behavior of CC1, we compared the genome sequences of the reference N2 and CC1 strains (McGrath et al. 2011) (wormbase.org), focusing primarily on variants in protein-coding sequences. This analysis led to the identification of missense variants in 8 protein-coding genes in CC1 compared with N2 (Supplementary Table 2). A subset of these changes is predicted to alter residues that may disrupt protein function (Fig. 2a; Supplementary Table 2) in a range of molecules including a predicted receptor guanylyl cyclase and a metabotropic glutamate receptor (Fig. 2a; Supplementary Table 2). Since the genomes of the strains used in our experiments likely differ from those of the reference strains, we verified the presence of these changes in the N2 and CC1 strains used in our behavioral assays. We confirmed the presence of 6 of these changes in our CC1 but not in our N2 strain, whereas 1 mutation was present in both strains (Supplementary Table 2).

The neuronal circuit driving negative thermotaxis includes the AFD, AWC, and ASI neurons, as well as the AIY and AIZ first layer interneurons (Mori and Ohshima 1995; Biron et al. 2008; Kuhara et al. 2008; Beverly et al. 2011; Hawk et al. 2018; Yeon et al. 2021). Cross-referencing with the CeNGEN neuron gene expression database (Taylor et al. 2021) indicated that *gcy-12*, *ufd-3*, *mgl-3*, and *scd-2* may be expressed in a subset of these neurons. However, since the gene expression profile of AFD is regulated by the temperature experience of the animal (Yu et al. 2014; Kobayashi et al. 2016; Harris et al. 2023), additional AFD-expressed genes may not have been identified in the CeNGEN analysis. We found that the negative thermotaxis phenotype of animals carrying predicted null mutations in each of the 6 identified genes in the N2 background did not phenocopy that of CC1 (Fig. 2b), suggesting that enhanced negative thermotaxis in CC1 could arise due to the specific missense mutation at an individual locus, variants in multiple genes, and/or other sequence changes in the CC1 genetic background.

### Identification of an RIL between N2 and CC1 with decreased negative thermotaxis behavior and locomotion defects

**Fig. 3.**
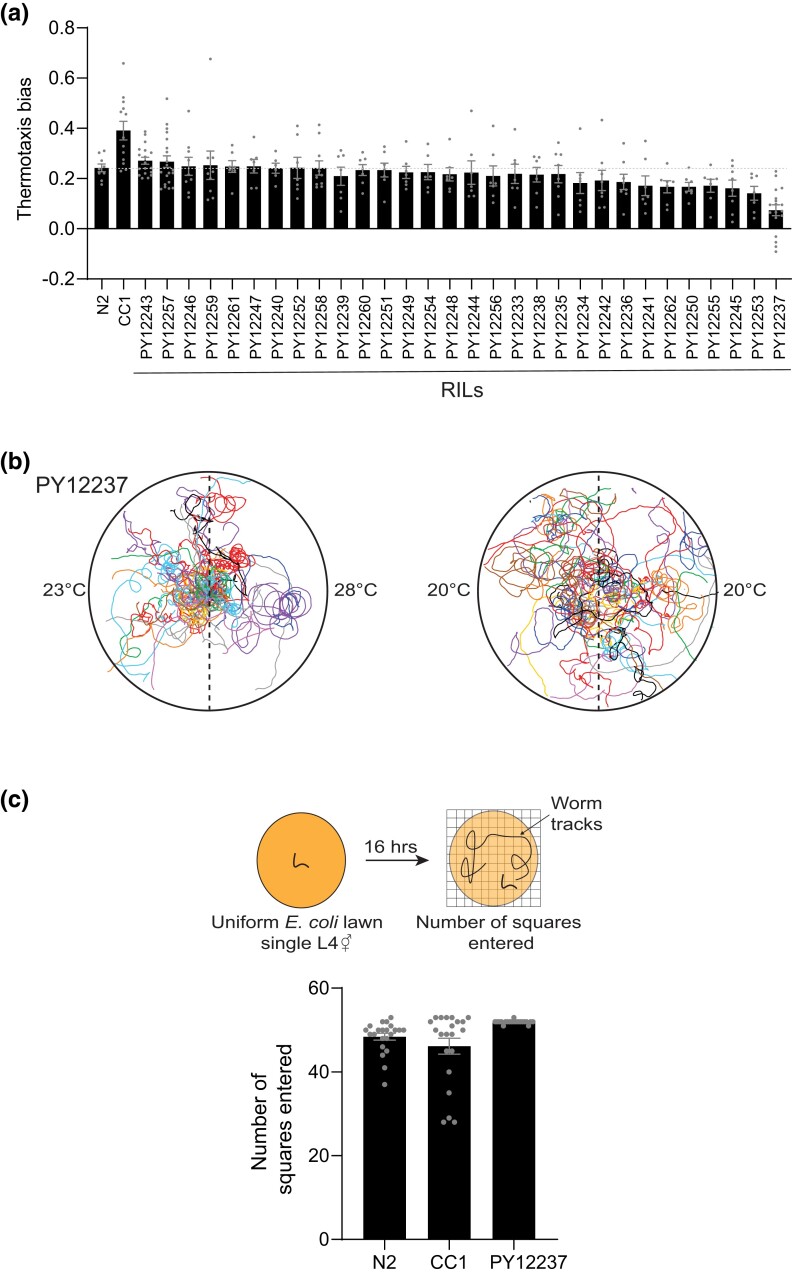
An RIL generated from interbreeding N2 and CC1 exhibits decreased negative thermotaxis and locomotory defects. a) Mean thermotaxis bias of animals from N2×CC1 RILs. Each dot is the thermotaxis bias of a single assay with 15 animals. Data shown are from at least 3 independent experiments. Animals were grown at 20°C and assayed on a 0.05°C/cm gradient from 23 to 28°C. Errors are SEM. b) Representative trajectories of PY12237 animals on a thermotaxis assay plate (left) and isothermal plate at 20°C (right). Trajectories of individual animals are color coded. c) Number of squares entered (top) by N2, CC1, or PY12237 animals during a grid exploration assay performed in the presence of bacterial food (see Materials and Methods) (Flavell et al. 2013). Each dot represents the movement of a single animal; assays were performed over multiple days. Errors are SEM.

To determine whether the variant(s) in CC1 driving improved negative thermotaxis can be genetically segregated, we generated a panel of 30 RILs from a cross between N2 and CC1 (Supplementary Fig. 1a) without performing behavioral selection. We subsequently examined their thermotaxis behavior. Although no RIL exhibited increased negative thermotaxis similar to CC1, we identified a strain (PY12237; Supplementary Table 3) that exhibited strong defects in negative thermotaxis (Fig. 3a).

Closer examination of the locomotory behavior of PY12237 showed that these animals circled extensively on the thermal gradient and did not exhibit directed movement toward the colder side of the gradient (Fig. 3b). This strain exhibited a similar locomotory phenotype on an isothermal plate (Fig. 3b) and also failed to move toward attractive odorants (Supplementary Fig. 1b). However, PY12237 animals explored robustly on bacteria-containing plates over a prolonged time period (Fig. 3c) (Flavell et al. 2013), indicating that the circling phenotype does not preclude exploratory behavior. Since neither N2 nor CC1 exhibited similar phenotypes (Fig. 1c; Supplementary Fig. 1b), we conclude that PY12237 contains spontaneously arising variant(s) driving locomotory and/or thermotaxis behavioral defects. Alternatively, these phenotypes are masked in the CC1 genetic background.

### Independently segregating variants contribute to the altered negative thermotaxis and locomotory behaviors of PY12237

**Fig. 4.**
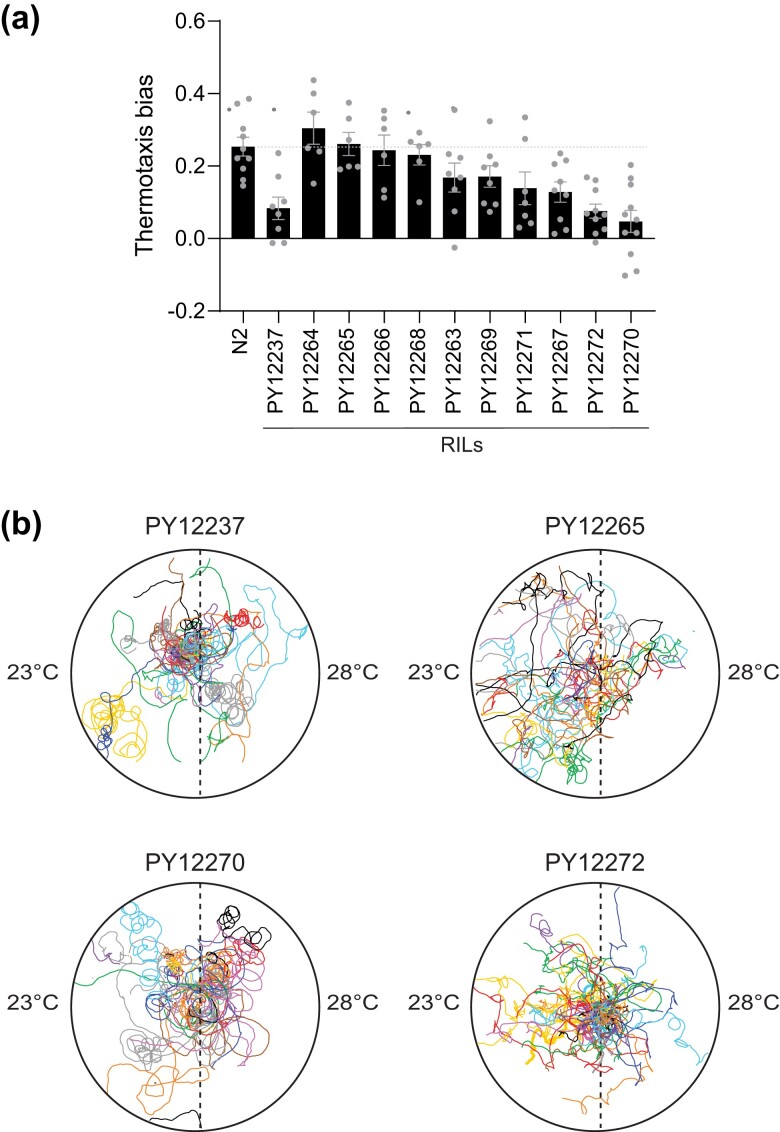
The thermotaxis behavioral defect of PY12237 is genetically separable from its locomotory defects. a) Mean thermotaxis bias of animals from strains generated via backcrossing PY12237 with N2. Each dot is the thermotaxis bias of a single assay with 15 animals. Data shown are from at least 3 independent experiments. Animals were grown at 20°C and assayed on a 0.05°C/cm gradient from 23 to 28°C. Errors are SEM. b) Representative trajectories of animals from the indicated strains on a thermotaxis assay plate in a single assay. Trajectories of individual animals are color coded.

We considered multiple hypotheses to account for the negative thermotaxis phenotype of PY12237. The defects in thermotaxis behavior could simply be a secondary consequence of the locomotory defects of this strain. Alternatively, the locomotory and thermotaxis phenotypes of PY12237 could arise due to either a single or multiple independent genomic changes. To distinguish among these possibilities, we crossed PY12237 to N2 and segregated 10 independent lines following self-fertilization for 8 generations. Two of these lines (PY12270 and PY12272) exhibited defects in negative thermotaxis similar to PY12237 (Fig. 4a). The examination of their locomotion showed that while PY12270 circled similarly to PY12237, PY12272 did not (Fig. 4b), indicating that the locomotory and thermotaxis defects are at least partly genetically separable. Consistently, PY12270 but not PY12272 also showed chemotaxis behavioral defects (Supplementary Fig. 2). PY12265, which performed robust negative thermotaxis (Fig. 4a), also did not exhibit circling on the assay plate (Fig. 4b). We infer that PY12237 contains at least 1 variant that results in locomotory defects with possibly secondary or parallel consequences on directed navigation behavior and an independently segregating variant that also affects negative thermotaxis.

### Spontaneously arising genetic variants in *ttx-1* contribute to altered locomotion and thermotaxis behavior in the CC1-derived PY12237 strain

**Fig. 5:**
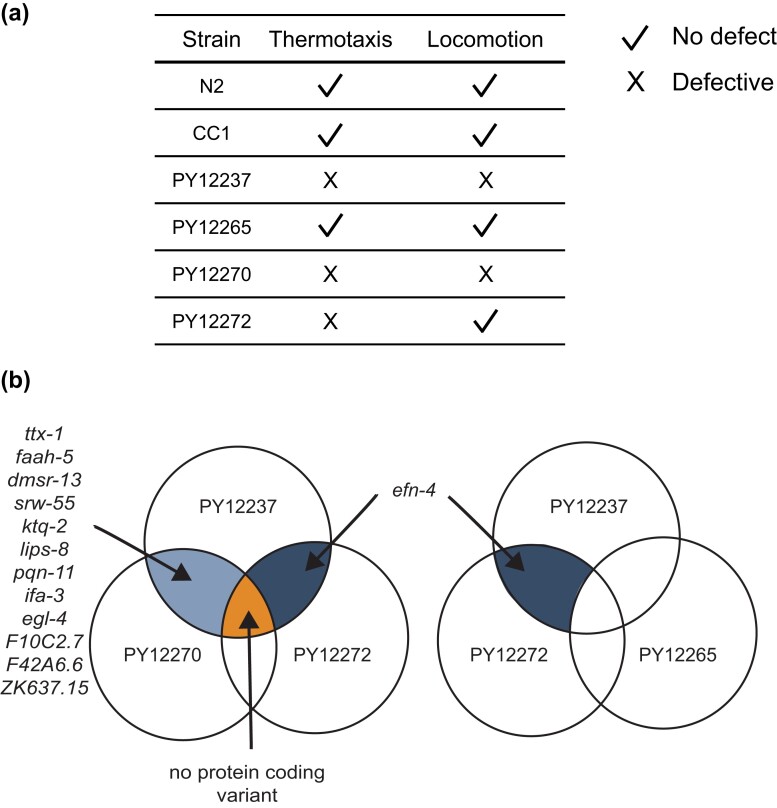
. Identification of shared protein-coding variants in RILs. a) Summary of thermotaxis and/or locomotory phenotypes of the indicated strains. b) Shared sequence variants present in the genomes of the indicated strains as identified via whole-genome sequencing. Only protein-coding variants are indicated (see Table 1).

To identify the causal variants regulating the locomotory and/or thermotaxis behavioral phenotypes of PY12237, we subjected PY12237, PY12270, and PY12272 to whole-genome sequencing. We also sequenced the genome of the PY12265 line that exhibits wild-type locomotion and negative thermotaxis (Fig. 4a–b). Since it is possible that these variants are also present in CC1 but are masked by epistasis, we additionally sequenced the genomes of our CC1 and N2 strains. We reasoned that variants causal to the circling behavior (with or without associated effects on thermotaxis) would be present in PY12237 and PY12270 but not in PY12265 or PY12272, whereas variants responsible for decreased negative thermotaxis alone would be present in PY12237 and PY12272 but not in PY12265 (Fig. 5a). The latter variants may or may not also be present in PY12270 since the locomotory defect of this strain precludes direct analysis of its thermotaxis behavior.

Focusing first on possible variants in protein-coding genes specifically affecting thermotaxis but not locomotory behavior, we identified a missense mutation only in *efn-4* present in both PY12237 and PY12272 but not in PY12270 or PY12265 (Fig. 5b; Table 1). This mutation was not present in additional RIL strains derived from PY12237 that exhibited normal thermotaxis or in CC1 (Table 1). *efn-4* encodes an ephrin homolog that has previously been shown to regulate neurite outgrowth in the AIY interneurons, a major component of the circuit driving thermotaxis (Chin-Sang et al. 1999; Schwieterman et al. 2016). The identified variant is a D47G missense mutation in a highly conserved residue in the β-strand C of EFN-4 predicted to be involved in the ligand–receptor tetramerization interface (Supplementary Fig. 3) (Chin-Sang et al. 1999). However, *efn-4(bx80)* null mutants exhibited robust negative thermotaxis (Supplementary Fig. 3), indicating that loss of function of *efn-4* is unlikely to underlie the thermotaxis defects of PY12237 and PY12272. No variants in protein-coding sequences were shared among PY12237, PY12270, and PY12272 (Fig. 5b). Since sequence changes present in PY12237 may also be present in CC1 but masked phenotypically, we also attempted to identify variants shared between CC1 and PY12237, PY12270, and PY12272 but not PY12265. However, no variants in protein-coding sequences were shared among these strains. We hypothesize that the thermotaxis defects of PY12272 may arise from noncoding variants or from changes at multiple loci.

**Table 1.** Summary of presence or absence of protein-coding variants in examined strains.

Gene	Encoded protein	Chr (position)*^a^*	Nucleotide change*^b^*	Protein change	Presence in					
					N2*^c^*	CC1*^c^*	PY12237	PY12265	PY12270	PY12272
*efn-4*	Ephrin	IV (565523)	A to G	Missense	No	No	Yes	No	No	Yes
*ZK637.15*	Uncharacterized protein	III (8921065)	A to C	Missense	No	No	Yes	No	Yes	No
*faah-5*	Amidase domain–containing protein	III (11887252)	C to T	Missense	No	No	Yes	No	Yes	No
*egl-4*	cGMP-dependent protein kinase	IV (1875890)	AGACTATGG AGA TAAGGA ACGTTTGGCT CAAGT to TCCCT	Indel	No	No	Yes	No	Yes	No
*F42A6.6*	DUF775 domain–containing protein	IV (3336613)	C to T	Missense	No	No	Yes	No	Yes	No
*dmsr-13*	GPCR	V (6819161)	A to C	Missense	No	No	Yes	No	Yes	No
*F10C2.7*	MFS domain–containing protein	V (12055146)	TC to GT	Missense	No	No	Yes	No	Yes	No
*srw-55*	GPCR	V (15939563)	A to G	Missense	No	No	Yes	No	Yes	No
*ttx-1*	OTX homeodomain protein	V (20064174)	T to C	Missense	No	No	Yes	No	Yes	No
*kqt-2*	Voltage-gated potassium channel	X (8245825)	C to T	Missense	No	No	Yes	No	Yes	No
*lips-8*	Lipase	X (14899594)	G to A	Missense	No	No	Yes	No	Yes	No
*pqn-11*	Prion-like domain–containing protein	X (15966176)	A to C	Missense	No	No	Yes	No	Yes	No
*ifa-3*	Intermediate filament protein	X (16269688)	C to T	Missense	No	No	Yes	No	Yes	No

wormbase.org.

Present in reference N2 and CC1 strains.

Reference N2 and CC1 strains; the absence or presence of the *ttx-1* variant was confirmed in the N2 and CC1 strains, respectively, used in this work.

**Fig. 6.**
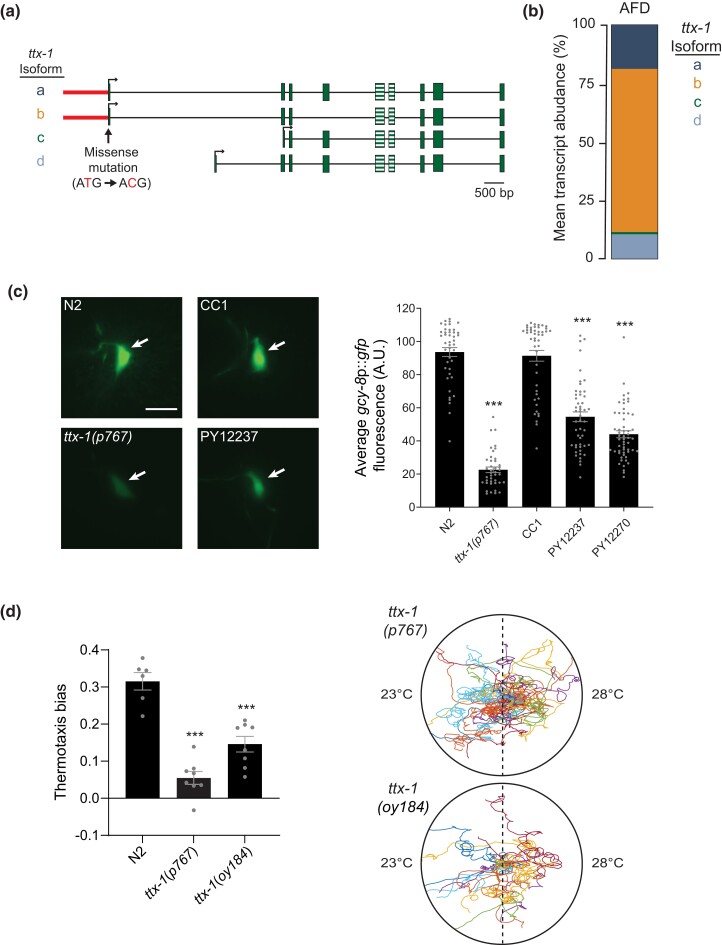
Sequence variants at the *ttx-1* locus in part underlie the locomotory and thermotaxis phenotypes of PY12237 and derived strains. a) Shown are the genomic structures of 4 alternatively spliced *ttx-1* isoforms. The missense mutation present in PY12237 and PY12270 but not in N2 or CC1 is indicated. Hatched boxes indicate sequences encoding the homeodomain. The presence of upstream chimeric and clipped reads is indicated in red. b) Diagrammatic representation of *ttx-1* transcript abundance in AFD from neuronal RNA-Seq data (Barrett et al. 2022). c) Representative images (left) and quantification of *gcy-8*p*::gfp* reporter fluorescence (right) in AFD in the indicated strains. Anterior is at the left in all images. AFD soma are indicated by arrows. Scale bar: 10 μm. Each dot is the fluorescence level in a single neuron. Animals from 2 independently generated transgenic lines were examined for each strain. Errors are SEM. *** indicates different at *P* < 0.001 from N2 (1-way ANOVA and Tukey's test). d) Mean thermotaxis bias (left) and representative trajectories (right) of N2 animals carrying indicated *ttx-1* alleles. The missense mutation shown in (a) was introduced into the *ttx-1* locus in the N2 background to generate *ttx-1(oy184)*. Each dot is the thermotaxis bias of a single assay with 15 animals. Data shown are from at least 3 independent experiments. Animals were grown at 20°C and assayed on a 0.05°C/cm gradient from 23 to 28°C. Trajectories of individual animals are color coded. Errors are SEM. *** indicates *P* < 0.001, compared with N2 (1-way ANOVA and Tukey's test).

We next identified changes underlying the locomotory and/or thermotaxis defect by identifying protein-coding variants present in PY12237 and PY12270 but not in PY12272 or PY12265 (Fig. 5b). Of the variants that fulfilled these criteria, we noted that 1 was associated with the *ttx-1* locus (Fig. 5b; Table 1). *ttx-1* encodes an OTX homeodomain transcription factor that serves as a terminal selector factor for specification of AFD thermosensory neuron fate (Satterlee et al. 2001). Interestingly, animals carrying loss-of-function mutations in *ttx-1* have been reported to not only exhibit strong thermotaxis behavioral defects but also to circle during directed navigation on bacteria-free plates (Ryu and Samuel 2002). Consistently, *ttx-1(p767)* exhibited atactic and circling behavior on thermal gradients similar to PY12237 and PY12270 animals (Fig. 6d).

We noted chimeric and clipped reads in a ∼1 kb region upstream of the *ttx-1* locus in both PY12237 and PY12270, suggesting a possible complex genomic structural change (Fig. 6a; Supplementary Fig. 4). The *ttx-1* locus is predicted to encode 4 distinct protein isoforms; each of these isoforms contains the conserved homeodomain but differ at their *N*-termini (Fig. 6a). In PY12237 and PY12270, we also identified a missense mutation converting the start codon of the *ttx-1.a* and *ttx-1.b* isoforms from Met to Thr; the *ttx-1.c* and *ttx-1.d* isoforms that include sequences encoding the homeodomain are predicted to be unaffected by this mutation (Fig. 6a). The *ttx-1*-associated variants were not present in either N2 or in CC1 (Table 1), suggesting that these changes arose spontaneously during the generation of PY12237. Recent bulk RNA-Seq of sorted individual neuron types showed that *ttx-1.b* is the predominantly expressed isoform in AFD (Taylor et al. 2021), and we previously showed that expression of this isoform alone can rescue AFD fate specification phenotypes (Satterlee et al. 2001). However, AFD also expresses *ttx-1.a* and *ttx-1.d* at lower levels (Barrett et al. 2022) (Fig. 6b), suggesting that the identified missense mutation is likely to decrease although not fully abolish *ttx-1* function.

TTX-1 regulates the expression of AFD-expressed terminal differentiation genes including the *gcy-8*-encoded receptor guanylyl cyclase (Satterlee et al. 2001). A *gcy-8*p*::gfp* reporter was expressed at significantly lower levels in PY12237 and PY12270 as compared with levels in N2 or CC1, although at levels higher than in a *ttx-1(p767)* loss-of-function mutant (Fig. 6c). Similar effects were observed in multiple transgenic strains carrying independent *gcy-8*p*::gfp*-containing extrachromosomal arrays. In addition, the introduction of only the missense mutation into the N2 background via gene editing [*ttx-1(oy184)*] resulted in thermotaxis and locomotory behavioral defects similar to those exhibited by PY12237 and PY12270 (Fig. 6d). We conclude that the missense mutation in *ttx-1*, possibly together with decreased expression due to the presumptive upstream genomic structural change, is the predominant driver of both the altered locomotion and negative thermotaxis behavior of PY12237 and PY12270 but that 1 or more independently segregating mutations likely contribute to decreased negative thermotaxis in PY12237 and PY12272.

## Discussion

Here, we identify spontaneously arising sequence changes that contribute to the thermotaxis and locomotory behavioral phenotypes of an RIL generated from intercrossing the N2 and CC1*C. elegans* strains. A missense mutation is predicted to affect 2 of 4 predicted isoforms of the *ttx-1* Otx homeobox gene, whereas a structural change lies in the upstream regulatory region of the *ttx-1* locus and may further affect *ttx-1* expression. Additional spontaneous sequence change(s) in PY12237 that segregate independently of the *ttx-1* variants may also contribute to its thermotaxis behavioral phenotype. These observations together with previous reports (Cook et al. 2006; Zhao et al. 2020) indicate that mutations arising *de novo* during the generation of RILs may complicate the analysis of QTLs contributing to phenotypic differences between strains.

Two lines of evidence support the notion that the identified spontaneous sequence changes at the *ttx-1* locus reduce protein function. First, the expression level of the AFD-specific and TTX-1-regulated *gcy-8* gene is decreased in RILs carrying the identified sequence changes, although the phenotype is weaker than in a strong *ttx-1* loss-of-function mutant. Second, while we were unable to fully resolve the complex sequence changes upstream of the *ttx-1* coding sequence, the introduction of the missense mutation present in the derived RILs into the *ttx-1* locus in N2 results in thermotaxis and locomotory phenotypes similar to those observed in PY12237 and PY12270. The missense mutation in *ttx-1* is predicted to specifically affect the *ttx-1.a* and *ttx-1.b* isoforms while sparing the remaining 2 isoforms. Further phenotypic analyses of animals expressing specific *ttx-1* transcripts in AFD may identify isoform-specific functions of this conserved protein.

Although large genomic structural rearrangements are often not maintained in populations due to negative fitness consequences (Lupski 2007; Katju and Bergthorsson 2013; Konrad et al. 2018), changes leading to gene duplication followed by functional divergence can be beneficial and maintained under positive selection (Taylor and Raes 2004; Kuzmin et al. 2022). *Caenorhabditis elegans* has been reported to have a high rate of spontaneous structural variants both within the laboratory and in wild strains (Maydan et al. 2010; Farslow et al. 2015; Konrad et al. 2018; Kim et al. 2019). The structural variant in the *ttx-1* upstream sequences may reduce *ttx-1* expression levels by affecting critical transcriptional regulatory sites. The divergence of transcriptional regulatory mechanisms can drive functional specialization of paralogous genes (Ohno 1970; Castillo-Davis et al. 2004; Huminiecki and Wolfe 2004; Li et al. 2005; Loker and Mann 2022). In rodents, Otx2 can functionally substitute for Otx1 in many tissues but the spatiotemporal expression pattern of these genes is distinct (Acampora et al. 1999; Suda et al. 1999) Similarly, while the 3 Otx type factors encoded by the *C. elegans* genome can functionally substitute for each other in examined contexts, these genes are expressed in different sensory neuron types in the adult (Satterlee et al. 2001; Lanjuin et al. 2003). Analyses of spontaneous mutations affecting regulatory sequences may allow for the identification of variants that drive the evolution of functional complexity in the wild.

This work began with the identification of enhanced negative thermotaxis behavior by the CC1 strain. However, we were unable to identify the causal genetic loci for this improved behavior. Thermotaxis behavior in *C. elegans* is influenced by multiple external and internal variables. For instance, navigation behavior toward the preferred temperature is regulated not only by the animal's recent temperature experience but also by the assay conditions including the steepness of the gradient as well as the animal's satiety state (Hedgecock and Russell 1975; Yamada and Ohshima 2003; Kodama et al. 2006; Ramot et al. 2008; Takeishi et al. 2020). While mutations in many individual loci including those required for AFD development or function can disrupt negative thermotaxis, improved behavior may arise from complex interactions among multiple loci (Gaertner et al. 2012). Loss-of-function mutations in individual genes in which we identified missense changes in CC1 did not exhibit altered thermotaxis behavior, raising the possibility that the specific missense mutations in 1 or multiple genes may underlie the observed enhanced negative thermotaxis. It is also possible that noncoding variants may contribute to the phenotypic difference. However, despite the relatively low sequence divergence between N2 and CC1, we did not identify an RIL among 30 examined strains that exhibited improved negative thermotaxis similar to CC1. It remains to be determined whether epistatic interactions among multiple loci affecting both coding and noncoding sequences underlie the thermotaxis behavioral phenotype of CC1.

While the analysis of spontaneously arising mutations influencing a particular trait allows for the identification of important contributing genetic loci, the presence of such changes even under highly regulated environments without obvious selection pressures on the trait under study suggests caution about assumptions regarding the genomic structures of RILs. Although many mutations arising *de novo* are unlikely to significantly affect the trait being studied, others may adversely influence the analysis of QTLs contributing to the phenotypic differences. The analysis of multiple RILs possibly generated from independent founder crosses as well as careful comparison of genomic sequences in RILs may address this concern.

## Supplementary Material

jkad186_Supplementary_Data

## Data Availability

Whole-genome sequences for all parent strains and RILs described in this work have been deposited at SRA (project accession number PRJNA987746). Underlying data for all behavioral experiments and variant analyses sequence files have been deposited to Figshare (https://doi.org/10.6084/m9.figshare.23565756). Custom Python scripts used for sequence analysis can be found at https://github.com/ptmcgrat/Sequencer/blob/master/NGS_Nematode.py. All other data necessary for confirming the conclusions of this article are represented fully within the article and its tables and figures. Supplemental material available at G3 online.
