# Supplementary material for: Identification of a spontaneously arising variant affecting thermotaxis behavior in a recombinant inbred Caenorhabditis elegans line
Source: G3 (Bethesda). 2023 Aug 12;13(10):jkad186. doi: 10.1093/g3journal/jkad186 (PMC10542565; doi:10.1093/g3journal/jkad186)
Supplement: jkad186_Supplementary_Data [file jkad186_supplementary_data.zip › Figure_S1_G3-2023-404443.pdf]

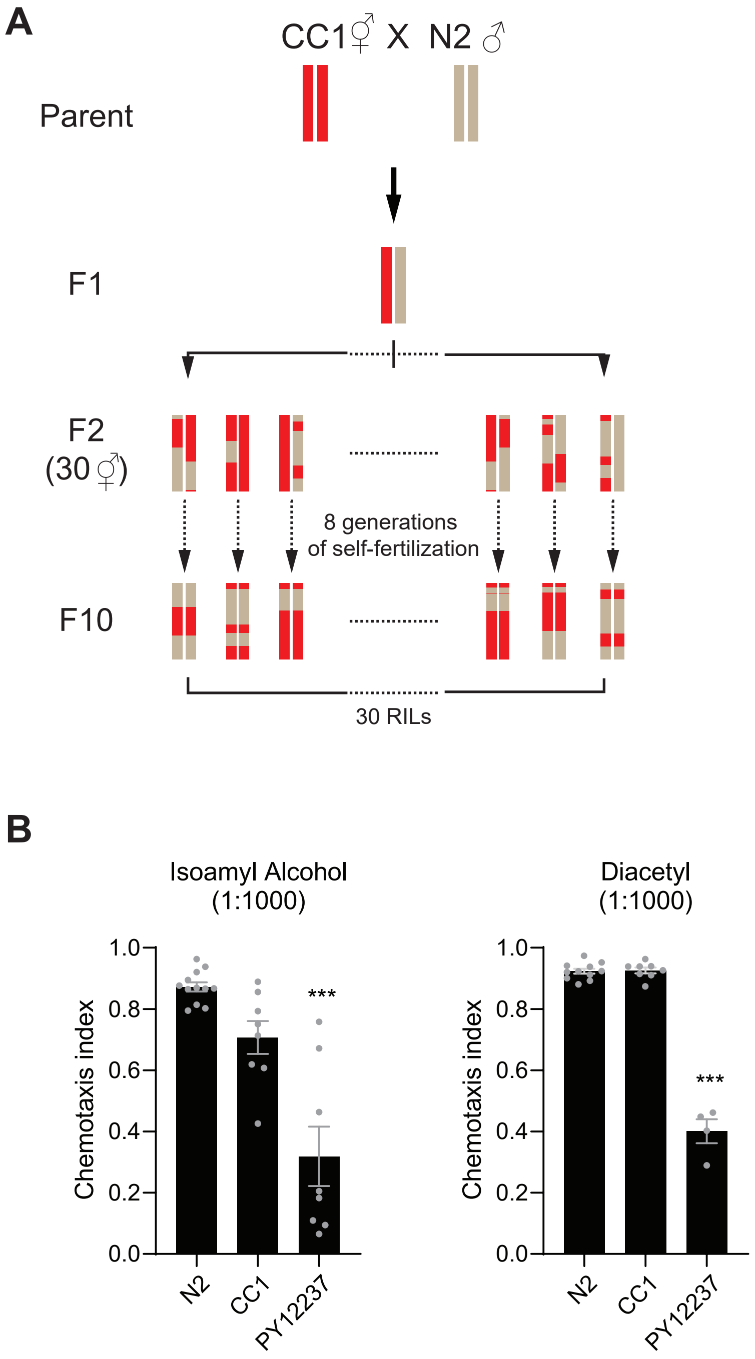

Figure S1. PY12237 exhibits defects in chemotaxis behaviors.

A) Scheme for the generation of RILs from a cross between N2 and CC1.

B) Chemotaxis responses of N2, CC1 and PY12237 animals to the indicated dilutions of the attractive odorants isoamyl alcohol and diacetyl. Each dot is the chemotaxis index of a single assay of ~150 animals. Data shown are from at least three independent experiments. Errors are SEM. *** indicates different from N2 at $p<0.001$ (one-way ANOVA with Bonferroni correction). N2 and PY12237 data are repeated in Figure S2.
