# Supplementary material for: Identification of a spontaneously arising variant affecting thermotaxis behavior in a recombinant inbred Caenorhabditis elegans line
Source: G3 (Bethesda). 2023 Aug 12;13(10):jkad186. doi: 10.1093/g3journal/jkad186 (PMC10542565; doi:10.1093/g3journal/jkad186)
Supplement: jkad186_Supplementary_Data [file jkad186_supplementary_data.zip › Figure_S2_G3-2023-404443.pdf]

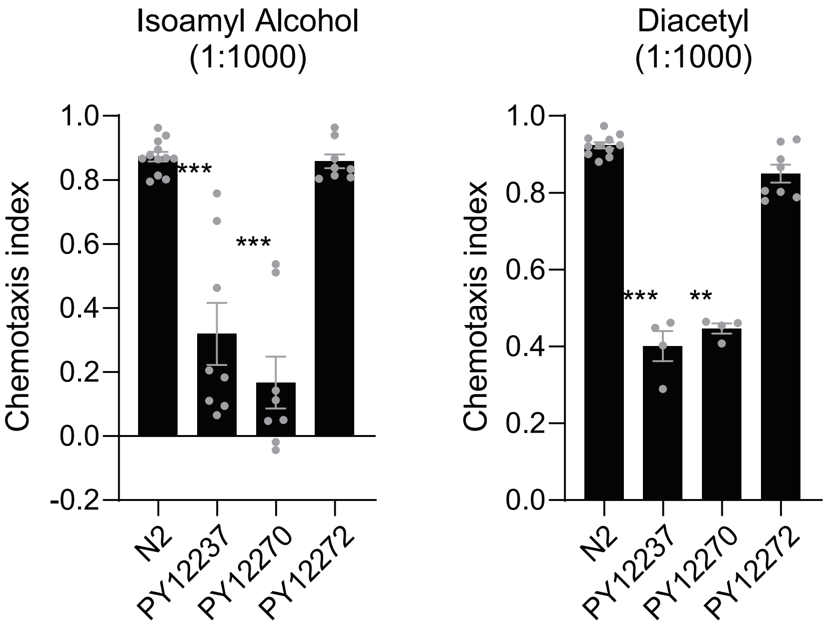

Figure S2. Chemotaxis behavioral phenotypes of strains derived from PY12237. Chemotaxis indices of the indicated strains to isoamyl alcohol and diacetyl. Each dot is the chemotaxis index of a single assay of ~150 animals. Data shown are from at least three independent experiments. Errors are SEM. ** and *** indicate different from N2 at p<0.01 and <0.001, respectively (one-way ANOVA with Bonferroni correction). N2 and PY12237 data are repeated in Figure S1.
