# Supplementary material for: Identification of a spontaneously arising variant affecting thermotaxis behavior in a recombinant inbred Caenorhabditis elegans line
Source: G3 (Bethesda). 2023 Aug 12;13(10):jkad186. doi: 10.1093/g3journal/jkad186 (PMC10542565; doi:10.1093/g3journal/jkad186)
Supplement: jkad186_Supplementary_Data [file jkad186_supplementary_data.zip › Figure_S3_G3-2023-404443.pdf]

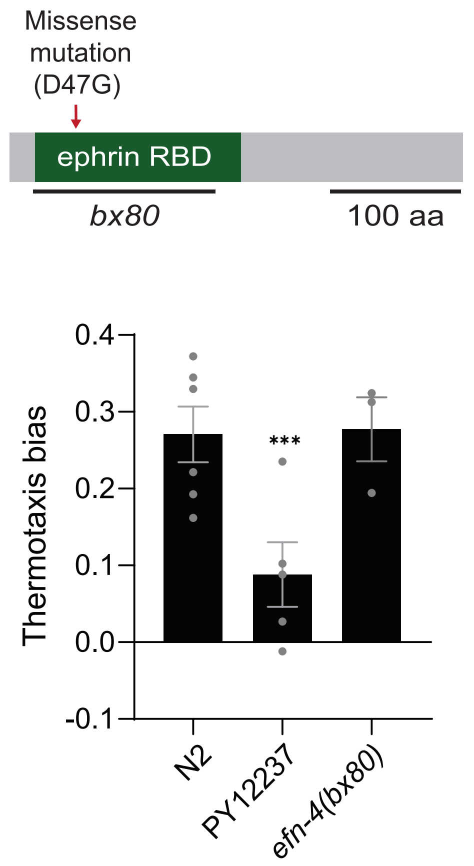

Figure S3. Phenotypic analysis of animals carrying variants in candidate protein-coding genes. (Top) Predicted domain structure of EFN-4 with the identity and location of the sequence change present in PY12237 and PY12272 but not in PY12270 or PY12265. The location of the predicted deletion in the *efn-4(bx80)* allele is also shown. RBD: receptor binding domain. (Bottom) Mean thermotaxis bias of the indicated strains. Each dot is the thermotaxis bias of a single assay of 15 animals; three independent assays. Animals were grown at 20ºC and assayed on a 0.05ºC/cm gradient from 23ºC-28ºC. Errors are SEM. *** indicates different from N2 at $p<0.005$ (one-way ANOVA and Tukey's test).
