# Supplementary material for: Identification of a spontaneously arising variant affecting thermotaxis behavior in a recombinant inbred Caenorhabditis elegans line
Source: G3 (Bethesda). 2023 Aug 12;13(10):jkad186. doi: 10.1093/g3journal/jkad186 (PMC10542565; doi:10.1093/g3journal/jkad186)
Supplement: jkad186_Supplementary_Data [file jkad186_supplementary_data.zip › Figure_S4_G3-2023-404443.pdf]

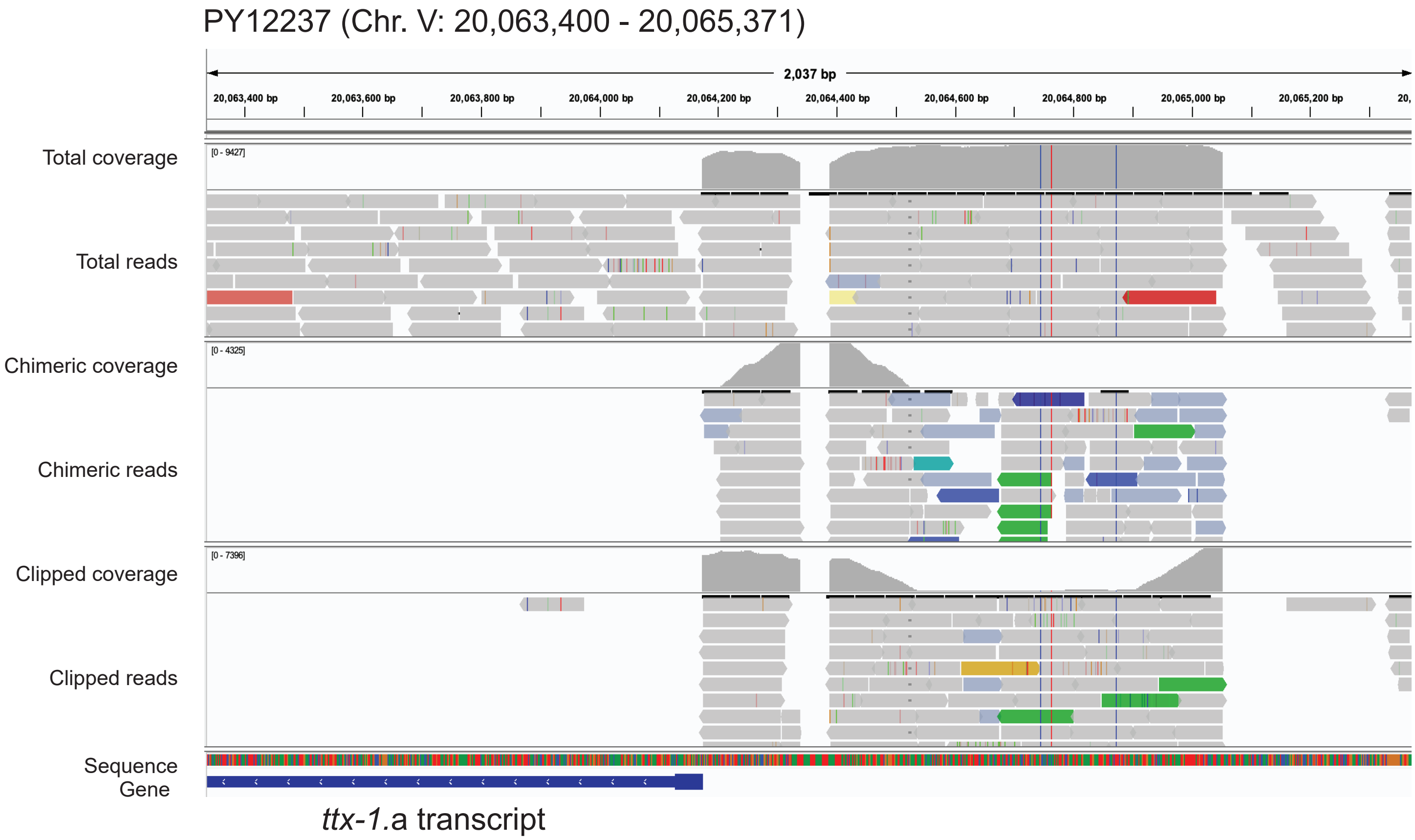

Figure S4. IGV view of the *ttx-1* genomic region (*Y113G7A.6*) in the PY12237 strain. The number of reads that mapped to the region directly upstream of the *ttx-1.a* transcript is shown in the top panel. Approximately 310x more reads mapped to a 900 bp region than expected. A large number of chimeric (middle panel - reads that map to two regions of the genome) and clipped reads (bottom panel – reads that only partially map to the genome) were also found in this region, suggesting the presence of a presumptive complex structural change that increased the copy number of this region. The N2 and CC1 strains did not show a similar increase in coverage.
