# Supplementary material for: Identification of a spontaneously arising variant affecting thermotaxis behavior in a recombinant inbred Caenorhabditis elegans line
Source: G3 (Bethesda). 2023 Aug 12;13(10):jkad186. doi: 10.1093/g3journal/jkad186 (PMC10542565; doi:10.1093/g3journal/jkad186)
Supplement: jkad186_Supplementary_Data [file jkad186_supplementary_data.zip › Table_S1_G3-2023-404443.docx]

**Table S1**: *C. elegans* strains examined in this work.

| **Strain** | **Source** | **Isolation location** |
| --- | --- | --- |
| N2 | CGC | Bristol, UK |
| TR389 | CGC; N2-derived | Madison, WI, USA |
| QX1211 | CGC | San Francisco, CA, USA |
| MY14 | CGC | Mecklenbeck, Germany |
| QG2813 | CaeNDR | Budderoo, Australia |
| CB4854 | CGC | Altadena, CA, USA |
| JU561 | CGC | Sainte Barbe, France |
| DR1350 | CGC | Pasadena, CA, USA |
| JU3280 | CaeNDR | Prague, Czech Republic |
| DL238 | CGC | Manuka, HI, USA |
| ED3040 | CaeNDR | Johannesburg, South Africa |
| LSJ1 | CGC – N2-derived | Bristol, UK |
| JU345 | CGC | Merlet, France |
| JU323 | CaeNDR | Merlet, France |
| ED3052 | CaeNDR | Ceres, South Africa |
| CB3198 | CGC | Pasadena, CA, USA |
| JU751 | CaeNDR | Le Perreux-sur-Marne, France |
| MY16 | CaeNDR | Mecklenbeck, Germany |
| JU775 | CGC | Lisbon, Portugal |
| CB4856 | CGC | Hawaii |
| ED3072 | CaeNDR | Limuru, Kenya |
| NIC523 | CaeNDR | Cadiz, Spain |
| JU2578 | CaeNDR | Bagnolet, France |
| JU1200 | CaeNDR | Dundonald, UK |
| PX178 | CGC | Eugene, OR, USA |
| KR314 | CGC | Vancouver, Canada |
| CB4852 | CGC | Unknown |
| JU1400 | CGC | Seville, Spain |
| AB1 | CGC | Adelaide, Australia |
| TR403 | CGC- N2 derived | Madison, WI, USA |
| CC1 | CGC – N2 derived | Bristol, UK |
