# Supplementary material for: Identification of a spontaneously arising variant affecting thermotaxis behavior in a recombinant inbred Caenorhabditis elegans line
Source: G3 (Bethesda). 2023 Aug 12;13(10):jkad186. doi: 10.1093/g3journal/jkad186 (PMC10542565; doi:10.1093/g3journal/jkad186)
Supplement: jkad186_Supplementary_Data [file jkad186_supplementary_data.zip › Table_S2_G3-2023-404443.docx]

**Table S2.** Protein coding variants in CC1 compared to N2 strains used in this work.

| **Gene** | **Predicted protein** | **Chr**  **(Position)^a^** | **Nucleotide**  **Change^b^** | **Protein**  **Change** | **Presence in N2 strain^c^** | **Presence in CC1 strain^c^** |
| --- | --- | --- | --- | --- | --- | --- |
| *C06B8.7* | SRCR domain-containing protein | V  (15501700) | C to G | missense | No | Yes |
| *ufd-3* | Ubiquitin fusion protein | II  (9942949) | G to A | missense | No | Yes |
| *scd-2* | Tyrosine kinase receptor | V  (6634939) | T to C | missense | No | Yes |
| *mgl-3* | Metabotropic receptor | IV  (5439388) | C to T | missense | No | Yes |
| *gcy-12* | Guanylate cyclase | II  (5316670) | C to A | missense | No | Yes |
| *Y102A11A.1* | Transmembrane protein | X  (2053691) | C to T | stop | No | Yes |
| *ZC247.1* | Uncharacterized protein | I  (10266450) | A to G | missense | Yes | Yes |
| *C17C3.15* | SPK domain containing protein | II  (5571605) | G to C | missense | ND | ND |

^a^wormbase.org

^b^As identified in the reference N2 and CC1 strains.

^c^Verified in the N2 and CC1 strains used in this work.
