# Supplementary material for: Identification of a spontaneously arising variant affecting thermotaxis behavior in a recombinant inbred Caenorhabditis elegans line
Source: G3 (Bethesda). 2023 Aug 12;13(10):jkad186. doi: 10.1093/g3journal/jkad186 (PMC10542565; doi:10.1093/g3journal/jkad186)
Supplement: jkad186_Supplementary_Data [file jkad186_supplementary_data.zip › Table_S3_G3-2023-404443.docx]

**Table S3.** Strains used in this work.

| Strain | Genotype/other name | Source | Figure |
| --- | --- | --- | --- |
| DA609 | *npr-1(ad609)* | CGC | Figure 1B |
| RB783 | *scd-2(ok565)* | CGC | Figure 2B |
| JT249 | *scd-2(sa249)* | CGC | Figure 2B |
| FX01766 | *mgl-3(tm1766)* | NBRP | Figure 2B |
| FX02915 | *ufd-3(tm2915)* | NBRP | Figure 2B |
| PY5693 | *gcy-12(ks100)* | CGC | Figure 2B |
| PY12232 | *Y102A11A.1(oy185)* | This paper | Figure 2B |
| VC2185 | *C06B8.7(ok2814)* | CGC | Figure 2B |
| PY12233 | RIL1 | This paper | Figure 3A |
| PY12234 | RIL2 | This paper | Figure 3A |
| PY12235 | RIL3 | This paper | Figure 3A |
| PY12236 | RIL4 | This paper | Figure 3A |
| PY12237 | RIL5 | This paper | Figure 3A, 3B, 3C, 4B, S1B, S2, S3 |
| PY12238 | RIL6 | This paper | Figure 3A |
| PY12239 | RIL7 | This paper | Figure 3A |
| PY12240 | RIL8 | This paper | Figure 3A |
| PY12241 | RIL9 | This paper | Figure 3A |
| PY12242 | RIL10 | This paper | Figure 3A |
| PY12243 | RIL11 | This paper | Figure 3A |
| PY12244 | RIL12 | This paper | Figure 3A |
| PY12245 | RIL13 | This paper | Figure 3A |
| PY12246 | RIL14 | This paper | Figure 3A |
| PY12247 | RIL15 | This paper | Figure 3A |
| PY12248 | RIL16 | This paper | Figure 3A |
| PY12249 | RIL17 | This paper | Figure 3A |
| PY12250 | RIL18 | This paper | Figure 3A |
| PY12251 | RIL19 | This paper | Figure 3A |
| PY12252 | RIL20 | This paper | Figure 3A |
| PY12253 | RIL21 | This paper | Figure 3A |
| PY12254 | RIL22 | This paper | Figure 3A |
| PY12255 | RIL23 | This paper | Figure 3A |
| PY12256 | RIL24 | This paper | Figure 3A |
| PY12257 | RIL25 | This paper | Figure 3A |
| PY12258 | RIL26 | This paper | Figure 3A |
| PY12259 | RIL27 | This paper | Figure 3A |
| PY12260 | RIL28 | This paper | Figure 3A |
| PY12261 | RIL29 | This paper | Figure 3A |
| PY12262 | RIL30 | This paper | Figure 3A |
| PY12263 | RIL5-1 | This paper | Figure 4A |
| PY12264 | RIL5-2 | This paper | Figure 4A |
| PY12265 | RIL5-3 | This paper | Figure 4A,4B |
| PY12266 | RIL5-4 | This paper | Figure 4A |
| PY12267 | RIL5-5 | This paper | Figure 4A |
| PY12268 | RIL5-6 | This paper | Figure 4A |
| PY12269 | RIL5-7 | This paper | Figure 4A |
| PY12270 | RIL5-8 | This paper | Figure 4A, 4B, S2 |
| PY12271 | RIL5-9 | This paper | Figure 4A |
| PY12272 | RIL5-10 | This paper | Figure 4A, 4B, S2 |
| PR767 | *ttx-1(p767)* | CGC | Figure 6C, 6D |
| PY12273 | *ttx-1(p767)*;  *oy719*[Ex(*gcy-8*p::*gfp)*] line-1 | This paper | Figure 6C |
| PY12274 | *ttx-1(p767)*;  *oy720*[Ex(*gcy-8*p::*gfp)*] line-2 | This paper | Figure 6C |
| PY12275 | CC1;  *oy721*[Ex(*gcy-8*p::*gfp)*] line-1 | This paper | Figure 6C |
| PY12276 | CC1;  *oy722*[Ex(*gcy-8*p::*gfp)*] line-2 | This paper | Figure 6C |
| PY12277 | RIL5;  *oy723*[Ex(*gcy-8*p::*gfp)*] line-1 | This paper | Figure 6C |
| PY12278 | RIL5;  *oy724*[Ex(*gcy-8*p::*gfp)*] line-2 | This paper | Figure 6C |
| PY12279 | RIL5-8;  *oy725*[Ex(*gcy-8*p::*gfp)*] line-1 | This paper | Figure 6C |
| PY12280 | RIL5-8;  *oy726*[Ex(*gcy-8*p::*gfp)*] line-2 | This paper | Figure 6C |
| PY12281 | *oy727*[Ex(*gcy-8*p::*gfp)*] line-1 | This paper | Figure 6C |
| PY12282 | *oy728*[Ex(*gcy-8*p::*gfp)*] line-2 | This paper | Figure 6C |
| PY12231 | *ttx-1(oy184)* | This paper | Figure 6D |
| EM305 | *efn-4(bx80)* | CGC | Figure S3 |
